# Single-digit arithmetic processing—anatomical evidence from statistical voxel-based lesion analysis

**DOI:** 10.3389/fnhum.2014.00286

**Published:** 2014-05-07

**Authors:** Urszula Mihulowicz, Klaus Willmes, Hans-Otto Karnath, Elise Klein

**Affiliations:** ^1^Division of Neuropsychology, Center of Neurology, Hertie-Institute for Clinical Brain Research, University of TübingenTübingen, Germany; ^2^Department of Diagnostics and Cognitive Neuropsychology, Institute of Psychology, University of TübingenTübingen, Germany; ^3^Section Neuropsychology, Department of Neurology, University Hospital RWTH AachenAachen, Germany; ^4^Knowledge Media Research Center, IWM-KMRCTübingen, Germany

**Keywords:** arithmetic, arithmetic facts, number processing, lesion analysis, stroke patients, fiber pathways

## Abstract

Different specific mechanisms have been suggested for solving single-digit arithmetic operations. However, the neural correlates underlying basic arithmetic (multiplication, addition, subtraction) are still under debate. In the present study, we systematically assessed single-digit arithmetic in a group of acute stroke patients (*n* = 45) with circumscribed left- or right-hemispheric brain lesions. Lesion sites significantly related to impaired performance were found only in the left-hemisphere damaged (LHD) group. Deficits in multiplication and addition were related to subcortical/white matter brain regions differing from those for subtraction tasks, corroborating the notion of distinct processing pathways for different arithmetic tasks. Additionally, our results further point to the importance of investigating fiber pathways in numerical cognition.

## Introduction

Despite numerous fMRI studies reporting the neural correlates of number processing (e.g., see Dehaene, 2009; Nieder and Dehaene, 2009 for reviews; see Arsalidou and Taylor, 2011 for a meta-analysis), there is still no agreement about the cognitive mechanisms involved in basic arithmetic, nor have its neural bases been delineated sufficiently. There is no consensus even with regard to single-digit operations, such as “2 × 3,” which are encountered frequently in every-day life and are assumed to be solved by retrieval from long-term memory without additional computation. These problems are commonly referred to as arithmetic facts, however a precise definition is rarely given (Domahs and Delazer, 2005).

Neuropsychological observations of double dissociations suggest that arithmetic facts are stored separately from other numerical information such as arithmetic concepts or procedures (Warrington, 1982; McCloskey et al., 1991b; McCloskey, 1992; Hittmair-Delazer et al., 1995; Delazer and Benke, 1997). The currently most influential model of numerical cognition, the Triple Code Model (TCM) by Dehaene et al. (2003) refers only to multiplication table facts, whereas addition is seen as a mixed operation, in which both direct and indirect processing pathways can be involved. In contrast, subtraction is assumed to rely essentially on magnitude processing, and it does not involve language-based processes (Lee and Kang, 2002). However, there is evidence that different arithmetic operations can be solved via diverse strategies (Lee, 2000) and considerable individual differences in strategy use have been reported (LeFevre et al., 1996a; Campbell and Xue, 2001; Thevenot et al., 2007). Self-reports suggest that the fact-retrieval strategy can be applied for easy items from all arithmetic operations, whereas it is much more often applied for multiplication (82%) and addition (75%) than for division and subtraction (cf. Campbell and Xue, 2001; Grabner et al., 2009). Thus, it has been suggested that single-digit addition tasks with sums smaller than ten, which do not involve “carrying,” can also be retrieved from memory directly (LeFevre et al., 1996b; Stanescu-Cosson et al., 2000; Klein et al., 2013). Findings by Thevenot et al. (2013) suggest that the retrieval strategy for addition tasks is more common in older as compared to younger subjects. Moreover, the bimodal distribution of reaction time (RT) data (Campbell, 2008) indicates that basic subtraction problems can also be solved by the retrieval strategy.

Another question concerns the interrelation of arithmetic operations. If different operations can be solved by the same strategy—do they share common neural representations? Results of neuroimaging studies provide evidence for separate representations (Arsalidou and Taylor, 2011; Rosenberg-Lee et al., 2011), however there is no comprehensive data from a group study in patients so far. The TCM (Dehaene et al., 2003) posits that arithmetic fact retrieval is subserved by left-hemispheric peri-sylvian and language areas. So far the TCM neither specifies in detail all language areas involved nor how they are connected for processing numerical information.

Therefore, the current study aimed to investigate single-digit arithmetic tasks in different arithmetic operations (i.e., addition, subtraction, multiplication) in a sample of 45 acute stroke patients in order to identify the brain structures crucial for their execution.

The variability of diagnostic tests and methodologies used in previous neuropsychological studies on single-digit arithmetic does not allow for direct comparisons. Single case studies have included patients with very divergent etiologies, such as closed head injury (e.g., McCloskey et al., 1991a), Fahr's disease (Delazer et al., 2004), dementia (e.g., Pesenti et al., 1994), or radiotherapy following leukemia (Hittmair-Delazer et al., 1995). These studies are informative as to the nature of the putative cognitive processes involved in relation to some processing model, yet they are not suited for determining critical brain areas involved in particular functions. Voxel-wise lesion-behavior mapping (VLBM) methods applied in an unselected stroke sample are considered a powerful approach to identify not only those brain structures which are “involved” in arithmetic fact retrieval but rather which are critically required for normal functioning. These methods implement inferential statistical analyses irrespective of clinical diagnoses or specified regions of interest. Moreover, these methods also allow to identify potential new brain areas in the network explored (Bates et al., 2003; Rorden et al., 2007). However, for valid VLBM-findings the examined sample should be unselected and the lesions should possibly cover the entire brain or at least large portions of it. Furthermore, because of neural plasticity, reorganization processes, and spontaneous recovery changes in shape, location, and functional integrity of brain tissue depend on the time post-stroke, potentially affecting the results of a VLBM analysis (Karnath and Rorden, 2012). To minimize these intervening sources, we decided to investigate patients only in the acute phase.

The aim of the current study was two-fold. First, we aimed at identifying brain structures critical for the execution of single-digit calculation. Based on the assumptions of the TCM and results of further neuroimaging studies, as summarized in a recent meta-analysis (Arsalidou and Taylor, 2011), we expected involvement of left perisylvian regions in arithmetic fact retrieval. Second, the study set off to systematically examine whether single-digit problems from different arithmetic operations are dependent on the same or different neural circuits.

## Methods

### Patients

Forty-five acute stroke patients, 21 left-hemisphere damaged (LHD), and 24 right-hemisphere damaged (RHD) participated in the study. This unselected sample comprised all patients consecutively admitted to the Center of Neurology at Tübingen University Clinic during 33 months, who met the inclusion criteria: MR or CT-documented cerebral stroke with cortical involvement, max. 14 days post-stroke, no previous lesions, no other neurological or psychiatric diseases, no substantial micro-angiopathy or white matter alterations, right-handedness, and German language as their mother tongue. Demographic and clinical data of all patients is presented in Table 1 and Supplementary Table 1. LHD patients were tested for language comprehension with the “Color-Figure” subtest items from the German adaptation of the Aphasia Check-List (ACL; Kalbe et al., 2005), and language production with the “Picture naming task” from the Aachener Aphasia-Bedside Test for acute patients (AABT; Biniek et al., 1992). Right-hemisphere patients underwent hemispatial neglect testing consisting of two cancellation tasks: “Letter Cancellation Task” (Weintraub and Mesulam, 1985) and “Bells test” (Gauthier et al., 1989), a copying task (Johannsen and Karnath, 2004), and a line bisection task (Heilman and Valenstein, 1979). Visual field deficits were assessed in all patients with a confrontation visual field test.

**Table 1:** **Demographic and clinical data of the left- and right-hemisphere damaged patients**.

		**LHD**	**RHD**
*n*		21	24
Sex (f/m)		15/6	11/13
Age (years)	Mean (SD)	61.6 (16.1)	61.0 (14.0)
Stroke type	Ischemic stroke	17	21
	Hemorrhagic stroke	4	3
Interval lesion onset to examination (days)	Mean (SD)	4.3 (1.9)	5.5 (2.8)
Interval lesion onset to imaging (days)	Mean (SD)	2.1 (2.0)	3.7 (3.5)
Education (years)	Mean (SD)	14.1 (4.4)	12.3 (4.5)
Contralateral paresis	% present	28.6	66.7
Visual field deficit	% present	23.8	20.8
Aphasia	% present	38.1	–
Neglect	% present	–	16.7

^*^Except for “Contralateral paresis” (χ^2^= 6.5, p = 0.011) there are no significant differences between the groups.

All patients gave their informed consent. The study was conducted in accordance with the ethical standards laid down in the 1964 Declaration of Helsinki and was approved by the ethics committee of the University Clinic Tübingen.

### Stimuli and procedure

Participants performed single-digit multiplication, addition, and subtraction tasks as part of a standardized neuropsychological battery examining number processing performance (Number Processing and Calculation (NPC) Battery; Delazer et al., 2003), also providing cut-off scores for impaired performance. In the NPC battery the three different arithmetic tasks constitute separate subtests. The standardized procedure requires that the testing per subtest is aborted after five consecutive incorrect or missing responses. Like for most cognitive neuropsychological assessment procedures, there is no time limit for answering the individual items. Self-corrections were allowed. Each calculation task was presented on a separate A4-sheet of paper (black digits printed on white paper, digit height: 7 mm). Sheets were aligned centrally on a table in front of the patient. Participants responded orally.

For the subsequent correlation of addition and subtraction tasks with lesion information we considered only those items of a subtest, which did not involve a carry or borrow operation (i.e., the sum was smaller than 10). Also, none of the items involved “0” or “1” as operands, because they are assumed to represent a distinct class of arithmetic problems implying rule-based processing (McCloskey et al., 1991a). With these item restriction criteria, the multiplication task comprised 36, the addition task 10, and the subtraction task 15 items.

To operationally determine impaired performance in the arithmetic tasks, we used cut-off criteria. For multiplication the cut-offs provided with the NPC-battery were used (cf. Delazer et al., 2003). The NPC-battery provides no separate cut-offs for addition items with sums below 10, or for subtraction items with minuends below 10. However, ceiling performance is expected in a healthy population. Thus, patients were considered to be showing a deficit in a given fact-retrieval task if their performance was below a mastery criterion computed by means of a procedure derived from criterion-referenced measurement. Using exact binomial (95%, i.e., 1 − α) confidence intervals computed for the relative frequency of items solved correctly, performance is considered to be in the mastery range if the upper bound of that interval is higher than some (high) criterion probability, e.g., *p_c_* = 0.95 or *p_c_* = 0.99. In case of very easy tasks like addition under 10, *p_c_* = 0.99 was considered to be appropriate. For the somewhat more difficult subtraction facts *p_c_* = 0.95 was employed.

In addition, to detect possible dissociations for individual patients' performance on different tasks, we performed specific, freely available single-case statistical tests for differences in level of performance (http://homepages.abdn.ac.uk/j.crawford/pages/dept/SingleCaseMethodology.htm; Deloche and Willmes, 2000; cf. Crawford and Garthwaite, 2005; Willmes, 2010) implementing operational definitions for different types of performance dissociations (classical and strong) as conceptually proposed by Shallice (1988).

### Lesion analysis

We used diffusion-weighted images for patients, who underwent MR-imaging within the first 48 h after stroke-onset (Weber et al., 2000), or T2-weighted fluid-attenuated inverse-recovery (FLAIR) contrast MR-imaging, if images were acquired later than that (Brant-Zawadzki et al., 1996; Noguchi et al., 1997; Ricci et al., 1999; Schaefer et al., 2002). If MR-images were not available, we employed CT-images. If several subsequent imaging data sets were available for the same patient, we chose the session acquired closest to the time of behavioral testing and providing the best imaging contrast for lesion demarcation.

Lesion borders were marked directly in the individual MR- or CT-scan using MRIcron software (www.mricro.com/mricron). Subsequently, both the anatomical scan and the lesion shape were mapped onto stereotaxic space using the “Clinical Toolbox” for normalization (Rorden et al., 2012; www.mccauslandcenter.sc.edu/CRNL/clinical-toolbox) implemented in SMP8 (www.fil.ion.ucl.ac.uk/spm). Some of the normalized lesion images had to be adjusted manually to the standard template by validating specific anatomical landmarks such as the basal ganglia. This was particularly the case in patients with extended hemorrhage, in which the normalization algorithm may lead to an unrealistic specification.

To investigate the relationship between lesion location and performance in the calculation tasks, we carried out separate VLBM analyses for each arithmetic operation and patient group (LHD or RHD, respectively) using the non-parametric Liebermeister test of the NPM software (Rorden et al., 2007) provided by the MRIcron package. For each voxel, the two subgroups of patients with resp. without a lesion in a given voxel were compared with regard to showing resp. not showing a deficit in the particular task. Because of the heavily left-skewed distributions of total scores correct per arithmetic operation item set, only this dichotomous performance measure was employed. Voxels damaged in at least one patient were included in the analysis. The results were corrected for multiple comparisons using a permutation-based family-wise error-correction approach with *p* < 0.05. Cortical and subcortical areas corresponding to voxels with a significant lesion-performance link were identified in the MNI-single subject space according to the Anatomical Automatic Labeling atlas (Tzourio-Mazoyer et al., 2002). White matter tracts were identified according to the diffusion tensor imaging (DTI)-based atlas by Catani and Thiebaut de Schotten (2012). In addition, probabilistic cytoarchitectonic maps of the white matter fiber tracts from the JuBrain atlas (Bürgel et al., 2006), implemented in the Anatomy Toolbox of the Juelich Research Center, were consulted to safeguard against possible differences in fiber tract labeling due to methodological differences in preparing different atlases.

## Results

**Figure 1:**
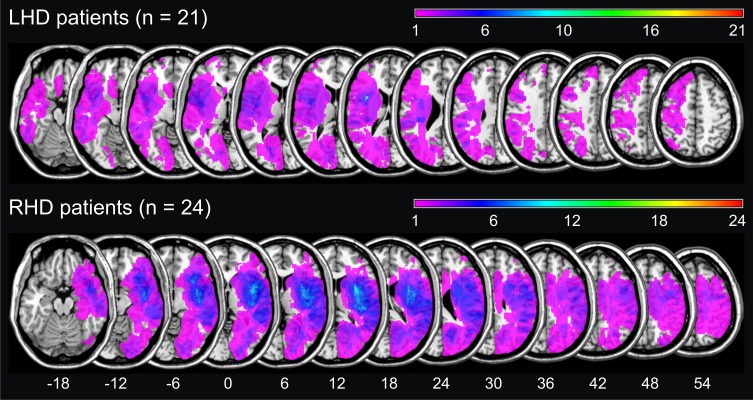
**Simple lesion-overlap for the LHD resp. the RHD patient group**. The number of overlapping lesions is color-coded with increasing frequencies from violet (*n* = 1) to red (*n* = maximum observed).

Figure 1 illustrates the conventional lesion density plot for all *n* = 45 patients with either LHD or RHD.

At the behavioral level, in the LHD group, 2 patients (L09, L13) showed a deficit in multiplication, 6 patients (L01, L04, L08, L13, L15, L16, L19) were impaired in single-digit addition, and 4 patients (L01, L04, L13, L15) in single-digit subtraction. In the RHD patient group, 3 patients (R09, R15, and R23) were impaired in single-digit multiplication. No patient with RHD was impaired in single-digit addition or subtraction. Nevertheless, overall performance was good, as is apparent from the raw data presented in Table 2. Testing was aborted due to five consecutive incorrect responses only in patient L01 for addition and subtraction, and patient L13 for multiplication.

**Table 2:** **Raw scores (number of items solved correctly) observed for the two patient groups in each arithmetic task**.

		**LHD (*n* = 21)**				**RHD (*n* = 24)**			
	**Items**	**Mean**	**Median**	***SD***	**Range**	**Mean**	**Median**	***SD***	**Range**
Addition	10	9.1	10.0	2.3	0–10	10.0	10.0	0.0	10–10
Subtraction	15	13.4	15.0	3.4	1–15	14.8	15.0	0.4	14–15
Multiplication	36	32.0	33.0	7.5	2–36	34.0	35.5	3.1	24–36

Additionally, dissociation in performance between the three arithmetic operations was observed. Details are given in the upper panel of Table 3.

**Table 3:** **Patients with dissociations between arithmetic and language tasks**.

**Dissociation**	**Classical^a^**	**Strong^b^**
Addition > Subtraction	L04	
Multiplication > Subtraction	L01	
Subtraction > Multiplication		L13
Multiplication > Addition	L01	
Addition > Multiplication	R09, R15	L13
Picture naming > Addition		L01
Addition > Picture naming	L16, L18	L10
Picture naming > Subtraction		L01
Subtraction > Picture naming	L10, L18	
Picture naming > Multiplication		L13

a *Only one function impaired and significantly poorer than the non-impaired function*.

b *Both functions impaired, but significantly different from each other (cf. Crawford and Garthwaite, 2005)*.

Several patients suffered from aphasia. To further explore the relationship of language and arithmetic deficits we tested for dissociations between performance in each arithmetic operation and the picture naming task. The results are presented in the lower panel of Table 3. In fact, there were patients (L09 for multiplication, L04 for subtraction) who were impaired on an arithmetic task despite no language deficit. However, these differences were not large enough to qualify as dissociation. All of the cases reported in Table 3 who performed significantly better on a language task than on an arithmetic task showed a strong dissociation.

**Figure 2:**
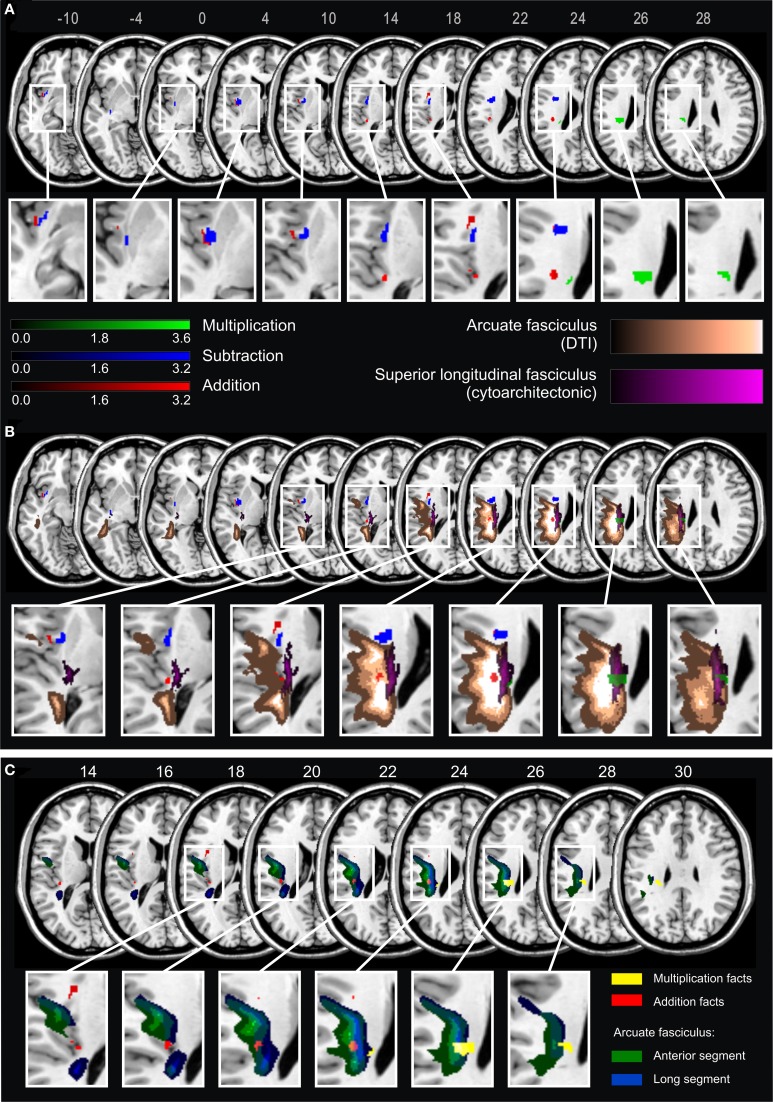
**(A)** Statistical voxel-wise lesion-behavior mapping (VLBM) analyses using the Liebermeister-test statistic for the dichotomous deficit yes/no-criterion in the three arithmetic tasks in 21 LHD patients. Plotted are voxels that survived a permutation-test based FEW correction at *p* < 0.05. Areas in red are associated with deficits in addition, in blue with subtraction, and in green with multiplication. Color bars indicate z-scores. MNI coordinates of transversal sections are indicated. **(B)** Significant lesion areas from panel **A** overlaid on white matter pathways: in orange the AF according to the DTI-based atlas by Catani and Thiebaut de Schotten (2012), and in violet the SLF according to the probabilistic cytoarchitectonic JuBrain atlas (Bürgel et al., 2006). The graded shadowing represents the probability of a given voxel belonging to the particular fascicle, where brighter color indicates higher probability. **(C)** Results of the VLBM analyses for multiplication facts (yellow) and addition facts (red) overlaid on two segments of the AF according to Catani and Thiebaut de Schotten (2012). The anterior segment of the AF is depicted in green and the long segment in blue. Note that the segments overlap partially. MNI coordinates of transversal sections are also indicated.

Results of the VLBM analyses are presented in Figure 2. In the LHD patient group, deficits in *single-digit multiplication* were significantly associated with a lesion in the superior longitudinal fascicle II (SLF II) according to the JuBrain atlas (Bürgel et al., 2006). This fiber bundle corresponds to the structure termed longitudinal segment of the arcuate fascicle (AF) according to the Atlas of Human Brain Connections (Catani and Thiebaut de Schotten, 2012) as apparent when overlaying these two pathway maps on the same template. In contrast, deficits in *single-digit addition* were significantly related to lesions of the insula, Rolandic operculum, Heschl's gyri, inferior frontal operculum, external capsule and the AF, but not the SLF II as described by the JuBrain atlas. For *single-digit subtraction* significant lesion-behavior correlations were found for insula, external/extreme capsule (EC/EmC)-system, and putamen. In the RHD patient group no significant correlations were observed^1^.

According to Catani and Thiebaut de Schotten (2012) the AF can be further partitioned into three segments: anterior, long and posterior segment. The long segment corresponds to the dorsal pathway directly connecting frontal, parietal and temporal cortices, whereas the anterior segment corresponds to more dorsal (superior) fibers connecting temporal and frontal regions via the parietal Geschwind's area. With regard to this partition of the AF the current results suggest that the part of the lesion map related to multiplication facts primarily comprised the long segment, while the map for addition mostly covered the anterior segment (Figure 2C). However, because the two segments of the AF partially overlap, a clear-cut distinction was not possible.

## Discussion

The objective of the current study was to identify brain regions critical for solving single-digit arithmetic tasks employing VLBM in a sizeable group of stroke patients. In particular, we aimed to explore differences in brain areas subserving this process in different arithmetic operations.

### Dissociation of operations

The present results show dissociations on the behavioral level, as well as distinct pathways for solving of single-digit tasks in different arithmetic operations. Deficits in multiplication were associated with more superior lesions in the SLF II according to the JuBrain atlas (Bürgel et al., 2006) than deficits in addition, although they both involved the AF according to the atlas by Catani and Thiebaut de Schotten (2012). In contrast, significant lesion maps for subtraction involved the external/extreme capsule-system and insular cortex. These results support the notion of relative distinctness of arithmetic operations, suggesting that even single-digit additions and subtractions are processed differently than multiplication table facts.

This finding is in line with the evidence from functional neuroimaging studies suggesting partly distinct processing patterns for different arithmetic tasks, as revealed by the meta-analysis of Arsalidou and Taylor (2011). However, Rosenberg-Lee et al. (2011) found different patterns of activations for addition, subtraction and multiplication tasks (carefully matched for difficulty and processing speed) only in the right hemisphere, whereas the left hemisphere activations were overlapping. In contrast, in the current study we observed differences between operations in a group of LHD patients, while no significant lesion-symptom correlations were found for the RHD patients group. Similar discrepancies have repeatedly been shown for language functions, where the regions of fMRI-activations in language processing did not exactly correspond to regions critical and necessary for the execution of a particular function (Binder et al., 2009). Another possible explanation for our divergent findings is the type of task employed, which was a verification task in the study by Rosenberg-Lee et al. (2011) in contrast to a production task in the current study.

The question about the source of discrepancies among arithmetic operations remains open. It has previously been argued that the neural correlates underlying the representations of multiplication table facts and subtraction problems diverge, because they rely on different solution strategies (Dehaene and Cohen, 1997; Lee, 2000; Tschentscher and Hauk, 2014). Although single-digit addition and subtraction problems analyzed in the current study are commonly considered all to be solved predominantly via retrieval from long term memory in a normal population (Campbell and Xue, 2001; Grabner et al., 2009), the results of Fayol and Thevenot (2012) and Barrouillet and Thevenot (2013) contradict this view: the reported solution times suggest that—in contrast to multiplication—even single-digit addition and subtraction might not be directly retrieved from memory. In the present study we measured patients' performance only with respect to accuracy. This approach is more common in neuropsychological testing, as RTs are highly variable and less informative in acute stroke patients. Thus, it remains ambiguous whether the observed dissociations result from distinct semantic representations or processing strategies underlying different arithmetic operations. Another source of differences might be the relative problem size of different arithmetic operations. While products of single-digit addition and subtraction task remain relatively small, single-digit multiplication yields much larger results. Several studies aiming at a comparison of different arithmetic operations have struggled with this issue (e.g., Dehaene and Cohen, 1991, 1997; Kazui et al., 2000; Lee, 2000; Van Harskamp and Cipolotti, 2001; Kawashima et al., 2004; Delazer et al., 2006; Ischebeck et al., 2006; Zhou et al., 2006). However, we are confident that for the specific question of the current study—single-digit tasks that could possibly be retrieved as rote facts—the issue of problem size is less dramatic than in case of complex calculation tasks.

Altogether, along with several neuroimaging studies (e.g., Arsalidou and Taylor, 2011; Prado et al., 2011; Fayol and Thevenot, 2012; Tschentscher and Hauk, 2014) our results challenge the traditional cognitive psychology models (Ashcraft, 1992; Campbell and Oliphant, 1992; Siegler and Shipley, 1995) assuming that single-digit addition, subtraction and multiplication are all solved through the use of very similar retrieval strategies.

### Disconnection as a source of arithmetic deficits

Interestingly, the main sites revealed by the VLBM analyses were located in the white matter of the brain. This may be explained by the fact that the cortical areas critical for arithmetic were to a large extent spared in our patient sample: most importantly, the angular gyrus (AG).

One of the claims of the TCM is the involvement of the left AG in language-based retrieval of arithmetic facts. This was confirmed in both neuroimaging studies in healthy participants (Delazer et al., 2003; Grabner et al., 2009; Zamarian et al., 2009), and several brain damaged patients (Cohen et al., 2000; Lee, 2000). Results of an fMRI study based on self-reports about calculation strategies further point to involvement of the left AG in fact-retrieval (Grabner et al., 2009). However, Zaunmüller et al. (2009) reported a diverging finding in a patient with severe multiplication fact-retrieval deficits, although his brain lesion did not involve the left AG (see also Van Harskamp et al., 2005 for a similar case). Another patient with preserved single digit multiplication, despite a lesion extending to the supramarginal gyrus and part of the left AG, was also described (Van Harskamp and Cipolotti, 2001). However, this latter patient suffered from dementia and brain atrophy.

Apparently, disruption of white matter tracts can be critical for arithmetic. This lesion locus seemed to be present in some single case studies (e.g., Van Harskamp et al., 2005; Zaunmüller et al., 2009) as well. For instance, a case of pure Gerstmann syndrome has been described after a subcortical lesion beneath, but sparing the AG itself (Mayer et al., 1999). In (Zaunmüller et al., 2009) both a lesion of the ventral external/extreme capsule system and the dorsal SLF II were reported but not discussed. While we agree with the original interpretation that a lesion of the basal ganglia may have added to the severe multiplication impairment of the patient, we want to suggest that even though the left AG was not affected by the lesion, this area was no longer connected to frontal areas such as Broca's area. Therefore, the observed disconnections of both dorsal and ventral fiber pathway systems may also account for the observed multiplication impairment.

Whereas cortical substrates of numerical cognition have been investigated extensively, white matter pathways mediating the complex, multimodal processes of calculation have not yet been attended to systematically. Individual differences in white matter integrity have been shown to predict arithmetic skills in children. In particular, arithmetic approximation skills correlated with fractional anisotropy in the anterior portion of the SLF (Tsang et al., 2009) and performance on a basic equations test correlated with fractional anisotropy in the left inferior lateral fascicle (Van Eimeren et al., 2008). In adolescents, fractional anisotropy and radial diffusivity of the left SLF, left superior corona radiata (as labeled by the JHU-atlas Mori et al., 2009 corresponding to the AF in Catani and Thiebaut de Schotten, 2012), and the left cortico-spinal tract correlated with performance on the math subtest of the Preliminary Scholastic Aptitude Test, which is a nationwide administered scholastic measure, including word problems, geometry, algebraic equations, and complex arithmetic (Matejko et al., 2013). In adults, a combined fMRI-DTI study revealed a correlation between gray matter activation during calculation (all four arithmetic operations taken together) and the microstructure of the adjacent white matter (Van Eimeren et al., 2010). Activation of the left AG correlated significantly with the fractional anisotropy values of left superior corona radiata. For small (product < 25) but not for large problem size items, the correlation was significant for the superior coronae radiatae bilaterally. Thus, some evidence pointing to the importance of white matter connections for arithmetic functioning has already been published.

### Relation to the TCM

The current results challenge the traditional psychological models of arithmetic but also the currently most popular neuropsychological model—the TCM (Dehaene et al., 2003).

Figure 1 reveals that the most important anatomical structures implied by the TCM, i.e., the left angular gyrus and the intraparietal sulcus (IPS) bilaterally, were not covered in our sample of LHD patients. Therefore, based on the premises of the modular cognitive neuropsychological TCM, one should not expect any of the patients to present with deficits in calculation, which were nonetheless observed in our sample.

One of the central postulates of the TCM is the general distinction between a mental number magnitude representation on the one side and verbally mediated fact retrieval processes on the other side. According to the TCM, arithmetic problems can be solved via two basic routes. First, rote and overlearned arithmetic facts can be retrieved from long-term memory without relying on quantity information via the so-called *direct* route. Alternatively, the arithmetic problem gets related to quantity information via the *indirect* semantic route in the bilateral intraparietal cortex and only then submitted to left perisylvian regions, in particular the left angular gyrus, and finally linked to a number word to be uttered (e.g., in case of more difficult tasks).

The TCM (Dehaene et al., 2003) does not yet specify the neuroanatomical connections between the proposed modules. The first attempt to systematically investigate white matter pathways involved in numerical cognition was made by Klein et al. (2013). The authors performed probabilistic DTI-based fiber tracking, taking as seed points areas of activation for easy and complex addition tasks (assumed to represent fact-retrieval and number magnitude-based processing, respectively). The resulting network included all major sites predicted by the TCM plus several other areas previously proposed as an amendment to the TCM. The authors identified two separate networks for easy and more difficult calculation, both involving dorsal (SLF) and ventral pathways (external/extreme capsule system) connecting frontal and parietal regions. Regions involved primarily in easy arithmetic tasks were connected predominantly by ventral fibers belonging to the middle longitudinal fascicle, converging in the sub-insular white matter near the claustrum as well as superior and medial part of the external and/or extreme capsule (Klein et al., 2013). These ventral pathways were also crucial for single-digit subtraction in the present study.

In contrast, deficits in addition and multiplication fact retrieval were associated with lesions of the dorsal pathways. In particular, lesion maps for multiplication facts involved major parts of the long segment of the AF (corresponding to the SLF II in other atlases), which constitutes a major, direct connection between temporal and temporo-parietal areas involved in arithmetic fact retrieval. For addition, the significant lesion map did not involve the SLF II bundle, although it also overlapped with the AF—most probably with its anterior part. This pattern is consistent with the TCM, which regards addition as a mixed operation, relying on both fact retrieval and (intraparietal) number magnitude processing. Accordingly, we observed that addition deficits may relate to lesions of both, the long segment and the anterior segment of the AF, which connects frontal with temporal parts indirectly via intraparietal areas (Catani et al., 2005). Multiplication, based primarily on rote fact retrieval, seems to rely rather on pathways directly connecting frontal and temporo-parietal areas.

Thereby, our results also demonstrate the importance of white matter pathway connections in the human brain. A recent atlas guiding glioma surgery suggests that most white matter pathways are not resectable (i.e., resection would most probably cause functional loss; Ius et al., 2011). Thus, for the interpretation of impairments in behavior observed in single-case studies or voxel-based lesion mapping studies not only gray matter lesions should be investigated but also disconnections of white matter fiber pathways.

### Limitations and future perspectives

Although the significant lesion maps for addition and subtraction deficits did not overlap, current statistical methods implemented in MRIcron do not yet allow for a direct (multivariate) comparison, which is a common problem in neuroscience research (Nieuwenhuis et al., 2011). The dissociation of operations can thus only be tested based on the behavioral data, where in fact significant differences were found.

However, at the behavioral level, three out of four patients impaired in subtraction also showed a deficit in addition. Since addition and subtraction are complementary operations, addition may be used as a back-up strategy to solve subtraction tasks and vice versa. In case one operation is impaired, the complementary back-up strategy is missing, thus making the other operation more error-prone. Alternatively, an association between impairments may be due to other reasons.

The cortical parts of the maps with significant lesion-performance association for addition and subtraction both encompass the insula, which has recently been suggested by Arsalidou and Taylor (2011) to be included in the TCM. These authors argue that the insula plays a rather non-specific role, being involved in switching between working memory and default states during problem solving, since in other studies the insula was associated with error processing (Hester et al., 2004) or the execution of responses (Huettel et al., 2001).

The current results suggest that solving single-digit arithmetic operations is subserved predominantly by the left hemisphere. This conforms with the majority of previous clinical evidence, on which the earlier version of the TCM is based (e.g., Dehaene and Cohen, 1997). In the fMRI literature left-lateralization of activation patterns is particularly evident for multiplication, whereas other arithmetic operations have been found to activate both hemispheres (e.g., Chochon et al., 1999; Zhou et al., 2007; Klein et al., 2010). Nonetheless, some of the RHD patients also did show deficits in solving arithmetic tasks: all of them only in multiplication.

Further, in the current study we used a standardized neuropsychological test. Like many other clinical assessments it only appraises accuracy and not solution times, because response times of acute patients are not as reliable and informative as in the healthy population. However, it is also possible that a lesion to a region causes slowing down of responses but no drop in accuracy.

In the current study we investigated a group of patients in acute stroke phase to avoid the influence of compensation and brain reorganization processes. The next step would be to investigate longitudinal aspects of brain damage at different stages of recovery from acute stroke to chronic phase. This would inform about the stability of observed structure-behavior correlations in light of spontaneous neural recovery and compensatory brain plasticity.

## Conclusions

In the present study, we provide first evidence from a voxel-based lesion mapping analysis in a sizeable group of acute stroke patients for distinct neural processing pathways in different arithmetic operations. We identified different white matter pathways that lead to arithmetic fact-retrieval deficits in different arithmetic operations when disrupted. Our findings contribute to reconciling diverging evidence about involvement of the AG in arithmetic fact retrieval, by showing that a disconnection of a cortical structure through a white matter lesion can be associated with deficits comparable to those after damage of the cortical structure itself. Our results also argue for further amendments of the anatomo-functional TCM, which does not yet provide inclusion of white matter interconnections of the multiple (cortical) processing modules it describes.

## Author contributions

Elise Klein, Klaus Willmes, and Hans-Otto Karnath designed the study. Urszula Mihulowicz conducted the study. Urszula Mihulowicz and Elise Klein performed the analyses. Urszula Mihulowicz prepared the figures. Urszula Mihulowicz, Elise Klein, Klaus Willmes, and Hans-Otto Karnath wrote the article.

### Conflict of interest statement

The authors declare that the research was conducted in the absence of any commercial or financial relationships that could be construed as a potential conflict of interest.

## Footnotes

^1^For the size of the current sample dichotomous analysis is the method of choice (Rorden et al., 2007). Yet, an analysis of continuous data (i.e., without cut-offs) using the Brunner-Munzel test revealed similar results; however, for addition the result was only marginally significant due to the overall small sample.

## Supplementary material

Supplementary Table 1 **Demographic and clinical data of all patients**.
