# Supplementary material for: Single-digit arithmetic processing—anatomical evidence from statistical voxel-based lesion analysis
Source: Front Hum Neurosci. 2014 May 7;8:286. doi: 10.3389/fnhum.2014.00286 (PMC4019872; doi:10.3389/fnhum.2014.00286)
Supplement: Supplementary Table 1 — Demographic and clinical data of all patients. [file DataSheet1.PDF]

Supplementary Table 1 | Demographic and clinical data of all patients.

No.	Side of lesion	Sex (f/m)	Age (Years)	Aetiology	Interval lesion onset to examination (days)	Interval lesion onset to imaging (days)	Education		Contralateral paresis	Visual field deficit	Picture naming	Token task	Neglect	Number span forwards	Number span backwards	Counting to 26	Magnitude comparison	Transoding: arabic numbers → token
							Years	Abitur										
L1	L	f	78	H	10	6		no	yes	no	-	2		4	2	-	+	-
L2	L	m	66	I	4	1		no	no	yes	+	1		6	4	+	+	+
L3	L	f	43	I	6	4		no	yes	no	+	0		4	2	+	+	+
L4	L	f	76	I	3	1		no	no	n.d.	+	2		n.a.	n.a.	+	+	-
L5	L	m	70	H	3	2		no	no	yes	+	0		5	3	+	+	+
L6	L	f	59	I	6	2		no	no	no	-	2		5	4	n.a.	n.a.	-
L7	L	f	53	I	6	4	12	no	yes	no	+	0		6	4	+	+	+
L8	L	f	74	I	2	1		no	no	no	-	3		4	2	-	+	+
L9	L	f	56	I	4	1		no	yes	no	n.a.	0		6	3	+	+	+
L10	L	f	19	I	2	0		yes	no	no	-	1		3	2	+	+	-
L11	L	f	78	I	4	1		no	no	no	-	1		5	4	+	+	+
L12	L	f	72	I	3	5		no	yes	yes	n.a.	n.a.		6	5	+	+	+
L13	L	m	50	I	5	1		no	no	no	-	3		n.a.	n.a.	-	+	-
L14	L	f	34	H	5	7	17	yes	no	no	+	0		6	4	+	+	+
L15	L	f	77	I	4	1	14	no	no	no	n.a.	3		5	2	+	+	-
L16	L	f	61	H	6	1	16	yes	no	no	-	2		6	4	+	+	-
L17	L	m	55	I	3	1	22	yes	no	yes	+	0		8	5	+	+	+
L18	L	m	63	I	4	1	15	no	yes	no	-	3		7	3	+	+	-
L19	L	f	54	I	4	1	11	no	no	no	+	1		4	4	+	+	+
L20	L	f	79	I	1	0	6	no	no	yes	+	1		n.a.	n.a.	+	+	+
L21	L	m	77	I	5	4	14	no	no	no	+	0		6	4	+	+	+
R1	R	m	50	I	4	0		no	yes	no			no	7	5	+	+	+
R2	R	m	54	I	3	5		yes	no	yes			no	6	4	+	+	+
R3	R	f	63	I	4	4	12	no	yes	no			no	5	4	+	+	+
R4	R	m	50	I	7	3		no	yes	no			yes	5	3	+	+	-
R5	R	f	68	I	5	1		no	yes	no			no	5	3	+	+	+
R6	R	f	72	I	10	7	8	no	yes	yes			no	4	4	+	+	+
R7	R	m	76	I	2	0	17	yes	yes	no			no	8	6	+	+	+
R8	R	m	59	I	2	6		yes	yes	no			no	6	4	+	+	-
R9	R	f	76	H	5	2		no	no	yes			yes	5	3	+	+	-
R10	R	f	38	I	7	5		no	yes	no			no	5	4	+	+	+
R11	R	m	49	I	7	2		no	no	yes			no	7	5	-	+	-
R12	R	f	68	I	7	2	13	no	yes	no			yes	7	4	-	+	+
R13	R	m	38	I	3	2	12	no	yes	n.d.			no	6	5	+	+	-
R14	R	f	42	I	5	6	15	no	yes	no			no	8	5	+	+	+
R15	R	f	76	I	6	1	5	no	yes	no			no	6	4	+	+	+
R16	R	m	36	H	3	4	20	yes	no	no			no	7	5	+	+	+
R17	R	m	83	I	8	6	13	no	no	n.d.			no	n.a.	n.a.	+	+	n.a.
R18	R	f	73	I	6	5	12	no	yes	no			no	n.a.	n.a.	+	-	+
R19	R	f	71	H	2	1	8	no	no	no			no	4	4	+	+	+
R20	R	M	63	I	11	16	15	no	yes	yes			no	6	n.a.	+	+	+
R21	R	f	56	I	2	1	15	no	no	no			no	5	2	+	+	+
R22	R	m	58	I	9	1	14	no	no	no			no	4	2	+	+	+
R23	R	m	66	I	3	1	16	yes	yes	no			yes	5	3	+	+	-
R24	R	m	79	I	10	7	3	no	yes	no			no	6	3	+	+	+

L - feft; R - right; f- female; m - male; I - Ischaemic; H - Hemorrhage; Abitur - German university entrance qualification; n.d. - not distinguishable; n.a. - not available; Token task: 0 / 1 / 2 / 3 - no / mild / moderate / severe impairment; “+” - intact; “-“ - impaired
